# High Phase Synchronization in Alpha Band Activity in Older Subjects With High Creativity

**DOI:** 10.3389/fnhum.2020.583049

**Published:** 2020-10-22

**Authors:** Sou Nobukawa, Teruya Yamanishi, Kanji Ueno, Kimiko Mizukami, Haruhiko Nishimura, Tetsuya Takahashi

**Affiliations:** ^1^Department of Computer Science, Chiba Institute of Technology, Narashino, Japan; ^2^AI & IoT Center, Department of Management and Information Sciences, Fukui University of Technology, Fukui, Japan; ^3^Department of Neuropsychiatry, University of Fukui, Fukui, Japan; ^4^Faculty of Medicine, Institute of Medical, Pharmaceutical, and Health Sciences, Kanazawa University, Kanazawa, Japan; ^5^Graduate School of Applied Informatics, University of Hyogo, Kobe, Japan; ^6^Research Center for Child Mental Development, Kanazawa University, Kanazawa, Japan; ^7^Uozu Shinkei Sanatorium, Uozu, Japan

**Keywords:** creativity, EEG, phase lag index (PLI), functional connectivity, synchronization

## Abstract

Despite growing evidence that high creativity leads to mental well-being in older individuals, the neurophysiological bases of creativity remain elusive. Creativity reportedly involves multiple brain areas and their functional interconnections. In particular, functional magnetic resonance imaging (fMRI) is used to investigate the role of patterns of functional connectivity between the default network and other networks in creative activity. These interactions among networks play the role of integrating various neural processes to support creative activity and involve attention, cognitive control, and memory. The electroencephalogram (EEG) enables researchers to capture a pattern of band-specific functional connectivity, as well as moment-to-moment dynamics of brain activity; this can be accomplished even in the resting-state by exploiting the excellent temporal resolution of the EEG. Furthermore, the recent advent of functional connectivity analysis in EEG studies has focused on the phase-difference variable because of its fine spatio-temporal resolution. Therefore, we hypothesized that the combining method of EEG signals having high-temporal resolution and the phase synchronization analysis having high-spatio-temporal resolutions brings a new insight of functional connectivity regarding high creative activity of older participants. In this study, we examined the resting-state EEG signal in 20 healthy older participants and estimated functional connectivities using the phase lag index (PLI), which evaluates the phase synchronization of EEG signals. Individual creativity was assessed using the S-A creativity test in a separate session before the EEG recording. In the analysis of associations of EEG measures with the S-A test scores, the covariate effect of the intelligence quotient was evaluated. As a result, higher individual S-A scores were significantly associated with higher node degrees, defined as the average PLI of a node (electrode) across all links with the remaining nodes, across all nodes at the alpha band. A conventional power spectrum analysis revealed no significant association with S-A scores in any frequency band. Older participants with high creativity exhibited high functional connectivity even in the resting-state, irrespective of intelligence quotient, which supports the theory that creativity entails widespread brain connectivity. Thus, PLIs derived from EEG data may provide new insights into the relationship between functional connectivity and creativity in healthy older people.

## 1. Introduction

Creativity is the fundamental ability to produce novel and appropriate work (Runco and Jaeger, 2012; reviewed in Sternberg, 1999; Amabile, 2018). The nature of creativity has been investigated for more than half a century from diverse standpoints, such as the assessment of creativity, the cognitive processes underlying creativity, and the physical and social environments that foster creativity (e.g., light, sound, color, space, and cultural factors) (reviewed in Sternberg, 1999; Amabile, 2018). Several early psychological studies concluded that creative activity is a high-level cognitive process produced by the integration of several mental processes and the gathering of useful knowledge (reviewed in Guilford, 1967). Indeed, recent neuroimaging studies have identified various brain regions as necessary for the emergence of creativity (reviewed in Arden et al., 2010; Dietrich and Kanso, 2010; Fink and Benedek, 2014; Boccia et al., 2015; Pidgeon et al., 2016; Stevens and Zabelina, 2019). Some of these studies have employed the electroencephalogram (EEG) to capture neural activity that reflects various brain functions specific to the different physiologically relevant frequency bands, by exploiting this method's advantageously fine temporal resolution (Klimesch et al., 2007). EEG studies have demonstrated alterations in frequency-band-specific neural activity during creative activity and have also predicted performance in tasks that test creativity (Grabner et al., 2007; Bazanova and Aftanas, 2008; Fink and Neubauer, 2008; Danko et al., 2009; Fink et al., 2009; Razumnikova et al., 2009; Volf and Tarasova, 2010; Rominger et al., 2019; reviewed in Fink and Benedek, 2014; Pidgeon et al., 2016; Stevens and Zabelina, 2019).

In EEG studies, conventional power spectrum approaches have demonstrated the involvement of brain region-specific and task-specific power enhancements in creative activity (Grabner et al., 2007; Danko et al., 2009; Fink et al., 2009; Volf and Tarasova, 2010; Rominger et al., 2019). For example, task-related, alpha-band power enhancements were reported in the frontal, parietal-occipital, and right-hemispheric regions (Grabner et al., 2007; Fink et al., 2009; Rominger et al., 2019), whereas Danko et al. (2009) reported enhancements of task-related beta- and gamma-band power in the temporal region. Additionally, Volf and Tarasova (2010) reported that in a visual creative thinking task, the highly creative group exhibited region-specific increases and decreases in theta and beta activities, as measured by event-related synchronization. The results of these studies support the assumption that activation of brain networks is a key feature of creative work.

Recently, alternatives to the power spectrum approach have appeared that use functional magnetic resonance imaging (fMRI) to investigate the role of patterns of functional connectivity in creative activity (Beaty et al., 2014, 2015, 2016, 2018, 2019; Maillet et al., 2019). Beaty et al. (2014, 2018) observed that when participants with high divergent-thinking ability engaged in creative tasks, increases occurred in the functional connectivity of the default network, the efficiency of the functional network topology, and the connectivity between the default network and other networks, such as the executive and salience networks. These interactions among networks play the role of integrating various neural processes to support creative activity and involve attention, cognitive control, and memory (Beaty et al., 2014, 2018; Benedek et al., 2014). As measured using fMRI, temporal interactions among these networks appear as dynamic transitions in functional connectivity, with distinctive region-specific and task-related patterns appearing during task performance (Beaty et al., 2015, 2016; Maillet et al., 2019).

Accumulating evidence suggests that high creativity leads to mental well-being in older individuals (Flood and Scharer, 2006; Flood and Phillips, 2007; Roskos-ewoldsen et al., 2008; Leon et al., 2014; Palmiero et al., 2014, 2017), which resulted in increased interest in the role of the neural activity underpinning creative functions in the aging process (Ueno et al., 2015; Privodnova and Volf, 2016; Privodnova et al., 2017; Privodnova, 2018; Adnan et al., 2019). Early studies on creativity and aging found an age-related decline in creative ability (Alpaugh and Birren, 1977; McCrae et al., 1987), while more recent findings suggest the long-term preservation of creative activity by the aging process (Roskos-ewoldsen et al., 2008; Leon et al., 2014; Palmiero et al., 2014, 2017). In older participants engaged in creative tasks, Privodnova and Volf (2016) and Privodnova et al. (2017) found task-related enhancements of theta activity in anterior brain areas in the initial stage of task performance, and in the final stage, task-related enhancement of alpha activity in the parieto-occipital area and reduction of beta activity in posterior areas. An fMRI study on creative activity and aging revealed that during creative tasks, the functional connectivity of the brains of older participants with high creativity increased significantly, including that of the default network (Adnan et al., 2019). Because this enhancement was not observed in younger participants (Adnan et al., 2019), these results support the idea of sparing of creativity in the aging process (Roskos-ewoldsen et al., 2008; Leon et al., 2014; Palmiero et al., 2014, 2017) and also imply age-related differences in the neural processes that produce creative activity.

Considering that the observed functional connectivities depend on frequency band (Stam and Van Dijk, 2002; Stam et al., 2007), neuroimaging modalities with high temporal resolution, such as EEG and magnetoencephalography (MEG), have been used effectively to capture the characteristics of brain connectivity. The recent advent of functional connectivity analysis in EEG/MEG studies has focused on the phase-difference variable because of its fine spatio-temporal resolution, which helps capture large-scale functional network structure across a wide range of frequencies (Stam and Van Dijk, 2002; Stam et al., 2007). For instance, a phase synchronization analysis, which is based on the phase lag index (PLI), possesses a higher spatial resolution than do conventional coherence measures based on the cross spectra of time series because it removes the influences of volume conduction on apparent synchronization (Stam et al., 2007), as well as an imaginary component of coherence expanded from the coherence measures (Nolte et al., 2004). Moreover, the PLI has a higher temporal resolution than the coherence measures, focusing on non-stationary and instantaneous phase dynamics instead (Stam et al., 2007). The PLI approach has been used to study both healthy (Palva and Palva, 2012; Roux and Uhlhaas, 2014) and pathological populations (Stam et al., 2006, 2009; Uhlhaas and Singer, 2006; Garrity et al., 2007; Orekhova et al., 2014; Engels et al., 2015; Ghanbari et al., 2015; Takahashi et al., 2017, 2018; Nobukawa et al., 2020). Regarding the moment-to-moment dynamical behaviors of EEG signals, which are produced by the mutual interactions among various brain regions, our previous study reported that the temporal complexity in this dynamical behavior reflects the ability of creativity even though in the resting state (Ueno et al., 2015). Consequently, the high spatio-temporal resolution of the phase synchronization approach is considered appropriate for the study of neural activity evoked by creative tasks and even of the resting-state neural activity, because these activities involve various brain regions and their functional interactions (Beaty et al., 2014, 2018; Benedek et al., 2014; Ueno et al., 2015). However, most of these studies were restricted to evaluating coherence measures (Jaušovec and Jaušovec, 2000; Razoumnikova, 2000; Razumnikova, 2004; Razumnikova et al., 2009; Zhou et al., 2018), and far fewer have evaluated the PLI (Rominger et al., 2019, 2020). Therefore, we hypothesized that the combining measuring method of EEG signals having high-temporal resolution and the phase synchronization analysis having high-spatio-temporal resolutions would foster new insight of functional connectivity regarding high creative activity of older participants. To this end, we aimed to evaluate the functional connectivity of the brain in the resting state in healthy older participants using the PLI extracted from EEG signals and to examine the relevance of this connectivity to individual creativity.

## 2. Materials and Methods

### 2.1. Participants

Twenty healthy, older participants were voluntarily recruited from a local community of Fukui prefecture (Ueno et al., 2015). The participants were medication-free, right-handed, and scored >28 on the Mini-Mental State Examination (Folstein et al., 1975). Exclusion criteria were as follows: major brain abnormalities found on conventional MRI; major medical or neurological conditions; history of alcohol or drug dependency; and internal diseases, including hypertension, hyperlipidemia, and diabetes mellitus.

To evaluate the creativity of each participant, we used the S-A creativity test, version C, by J. P. Guilford (Society For Creative Minds, 1969). This is an established test for the evaluation of creativity that assesses the capacity for divergent thinking. In the present study, a Japanese version was used. The participants' creativity was assessed in a separate session before the EEG recording session. The S-A test comprises three types of tasks: the first measures the ability to generate unique ways of using common objects, the second quantifies the ability to imagine desirable functions of ordinary objects, and the third measures the ability to imagine the consequences of an unforeseen event. In each task, the participants produce as many answers as possible in <5 min. The results of these tasks were assessed using four criteria: fluency, flexibility, originality, and elaborate thinking. In this study, we used the sum of these four scores to represent creative ability. The four abilities assessed using the S-A test correspond to those measured using the Torrance test of creative thinking (Torrance, 1990). In the present study, participants were divided by the median of the S-A score distribution into two groups of 10 subjects each: high-creativity (high-scoring) and low-creativity (low-scoring) groups. In addition to creativity, intelligence quotient (IQ) was measured using a general test based on the Wechsler Adult Intelligence Scale-III (Wechsler, 1997). We used the full-scale IQ score in this study. Detailed information on the participants, their IQs, and their creativity scores are shown in Table 1. The sex ratios and age distributions of the groups were matched. The IQ scores of the high-creativity group were higher than those of the low-creativity group. The participants' scores in each of the four task criteria, i.e., fluency, flexibility, originality, and elaborate thinking, are represented in the Supplementary Material.

**Table 1:** Physical characteristics of the high- and low-creativity groups (values are mean [SD]).

	**High-creativity group (*n* = 10)**	**Low-creativity group (*n* = 10)**	**p-values**
Female/male	5/5	6/4	0.65
Age, years	72.2(4.1)	70.7(5.3)	0.54
S-A creativity test	113.1(17.3)	71.5(11.0)	< **0.01**
IQ	115.2(11.0)	104.6(7.2)	**0.02**

*For clarity, any comparisons yielding a p < 0.05 are shown in bold*.

All participants provided informed consent before the initiation of the study. The study protocol was approved by the ethics committee of the University of Fukui. All procedures of this study were performed in accordance with the Declaration of Helsinki.

### 2.2. EEG Recording

Participants were comfortably seated in a chair in an electrically shielded, soundproof, and light-controlled room. During EEG recording, they were in a state of wakefulness with their eyes closed for at least 3 min (Ueno et al., 2015). The EEG data were recorded with a 21-channel system (EEG–1514, Nihon-Koden, Tokyo, Japan) at 19 electrode sites assigned using the International 10–20 System, as follows: Fp1, Fp2, F3, F4, C3, C4, P3, P4, O1, O2, F7, F8, T3, T4, T5, T6, Fz, Cz, and Pz, with the two ear lobes jointly forming the reference. Eye movements were monitored using additional electro-oculographic channels.

The EEG signals were recorded with a sampling frequency of 500 Hz, a 1.5–60 Hz bandpass filter, and a time constant of 0.3 s. Because the bandpass-filtered data contained little line noise at 60 Hz, a notch filter was not applied. Electrode impedances were all <5 *k*Ω. Artifacts, including eye movements, blinks, and muscle activity, were manually excluded.

### 2.3. Phase Lag Index

To estimate functional connectivity, we utilized the PLI (Stam et al., 2007). The EEG signal was filtered into five pass-bands (2–4 Hz for the delta band, 4–8 Hz for the theta band, 8–13 Hz for the alpha band, 13–30 Hz for the beta band, and 30–60 Hz for the gamma band). At time *t*, electrode *a* is represented by the phase ϕ_*a*_ (*t*) and the amplitude *A*_*a*_ (*t*) via the Hilbert transform. The difference in the phase Δϕ_*ab*_ (*t*_*i*_) between the observed complex signals with two different electrodes *a* and *b* at some time *t*_*i*_ (*i* = 1, 2, …, *T*, where *T* is the length of one epoch of data points) is written as follows:

(1) $$\begin{array}{r} \Delta \phi_{ab}\;\left(t_{i}\right)=\phi_{a}\;\left(t_{i}\right)-\phi_{b}\;\left(t_{i}\right)\;, \end{array}$$

and

(2) $$\begin{array}{r} \Delta \phi_{\text{mod}}\;\left(t_{i}\right)=\Delta \phi_{ab}\left(t_{i}\right)\text{ mod }2\pi \;. \end{array}$$

From Equation (2), we obtain |Δϕ_mod_ (*t*_*i*_)| ≤ π. The PLI between the two observed points *a* − *b* is defined as

(3) $${\text{PLI}}_{ab}=\left|\frac{1}{T}\sum_{i=0}^{T}\text{sign}\left(\Delta \phi_{\mathrm{mod}}\left(t_{i}\right)\right)\right|,$$

where the PLI in Equation (3) becomes ≈ 1 in the synchronous state and ≈ 0 in a de-synchronous state. From Equations (1) and (2), the PLI is 0 when two signals observed at different points have a common source because Δϕ_*ab*_ (*t*_*i*_) is 0, and Δϕ_mod_ (*t*_*i*_) = 0. In addition, an observation *b* at a point located on the opposite side of the electric dipole that models the source of the signal *a* has Δϕ_*ab*_ (*t*_*i*_) = π in Equation (1). In our analysis, the PLI_*ab*_ values were averaged across epochs and the epoch length of the EEG data was 5 s (Takahashi et al., 2017, 2018). In calculating the PLIs from the EEG data after frequency-band isolation, we used HERMES as a toolbox for the EEG/MEG analyzer in MATLAB® by MathWorks (Niso et al., 2013).

We considered the average of all PLIs sharing a given electrode *a*, which is called the node degree of *a*, and is widely used for the analysis of functional connectivity (Takahashi et al., 2017, 2018; Nobukawa et al., 2019):

(4) $$\begin{array}{r} {\text{ND}}_{a}=\frac{1}{K-1}\overset{K}{\underset{b=1,b\neq a}{\sum }}\;{\text{PLI}}_{ab}\;, \end{array}$$

where *K* in Equation (4) represents the total number of electrodes, and here *K* = 19.

### 2.4. Power Spectrum

In addition to the functional connectivity estimation by PLI, we computed the power spectra of our EEG signals. In this analysis, we estimated the power spectral density (PSD) in dB/Hz using Welch's method with a Hanning window function having a width of 2.0 s. In addition to the PSD, we computed the relative powers of our five bands. For this analysis, we used the Signal Processing toolbox in MATLAB® by MathWorks.

### 2.5. Statistical Analyses

For the electrode-pair-wise group comparisons of the PLIs, we used a *t*-statistical analysis controlled by the Benjamini—Hochberg false discovery rate (FDR) procedure (Benjamini and Hochberg, 1995). Consequently, *t*-scores adjusted to *q* < 0.05 were used for the PLI analysis (855 *p*-values: 171 electrode pairs × 5 frequency bands). For the electrode-wise group comparisons of the PSDs, a *t*-statistical test with a Benjamini—Hochberg FDR correction was also used. As in the PLI analysis, *q* < 0.05 was applied (1,121 *p* values: 59 frequency points [2–60 Hz; width of a frequency bin is 1.0 Hz] × 19 electrodes).

To test for group differences in the relative powers and in the node degrees calculated from the PLIs, we used a mixed-design analysis of variance (ANOVA) with group (high creativity vs. low creativity) scores as the between-participants factor and nodes (19 electrodes) as the within-participants factor. As shown in the Supplementary Material, we confirmed that the covariate effect of IQ and age is not significant. We applied the Greenhouse-Geisser adjustment to assess the degrees of freedom. *Post hoc* *t*-tests of electrode-wise comparisons of node degree adjusted for FDR correction (*q* < 0.01, 0.05) were used to identify the sources of the significant main and interaction effects of group. In addition, we performed a correlation analysis of the creativity scores vs. node degree using Pearson correlation coefficients.

## 3. Results

### 3.1. Power Spectrum Analysis

**Figure 1:**
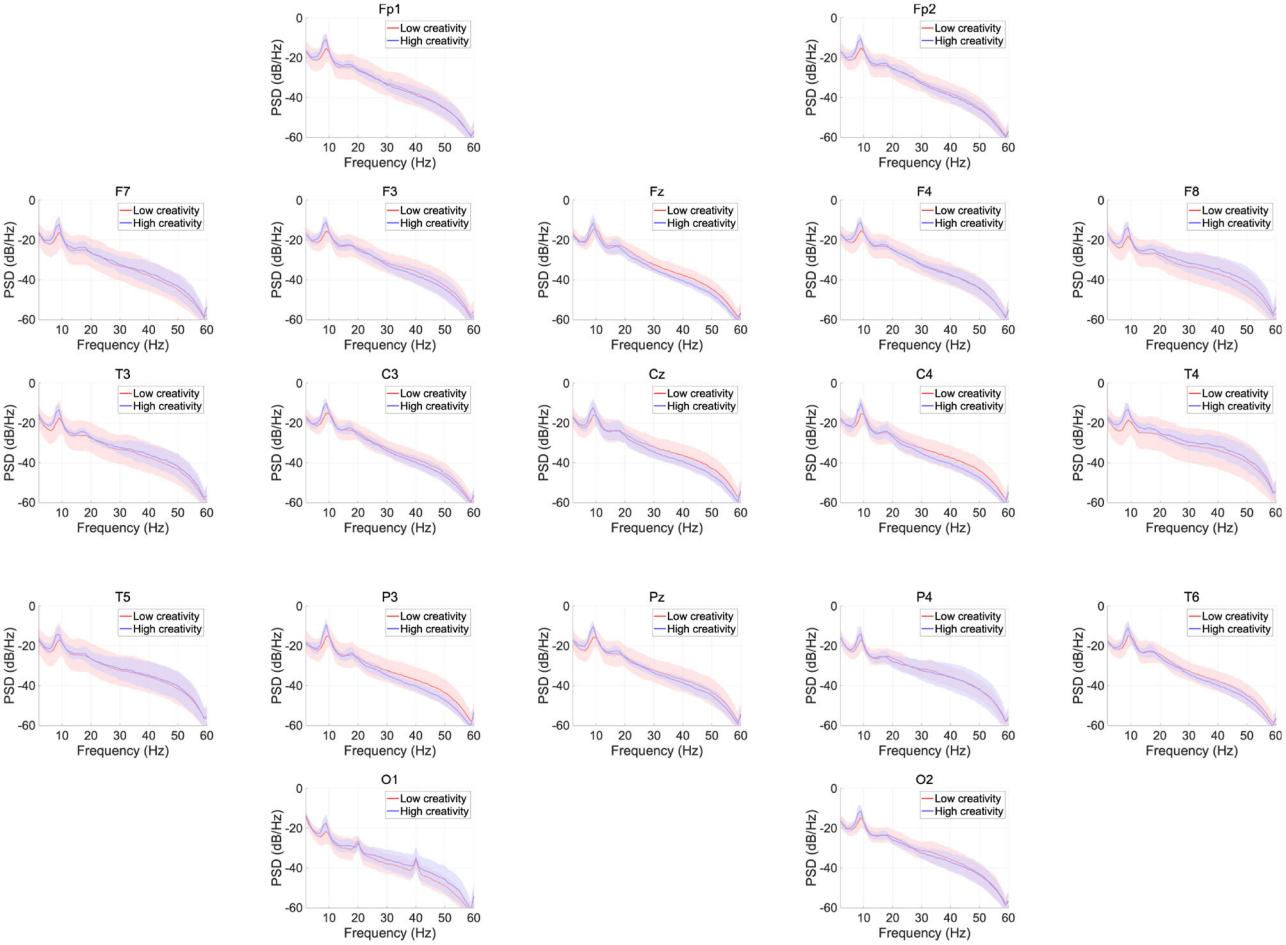
EEG power spectral density (PSD) for the high- and low-creativity groups. Solid lines and shaded areas indicate mean and standard deviation in each group. We observed no significant differences after adjustment for false discovery rate (FDR) with *q* < 0.05.

Figure 1 shows the EEG PSD data for the high- and low-creativity groups. In this power evaluation, we did not observe significant differences after adjustment for FDR at *q* < 0.05. Table 2 summarizes the results of mixed-design ANOVA, for relative power in each band. The results show that no significant group effect or group × node interaction was identified in any band.

**Table 2:** Assessment of relative powers in each frequency band comparing high- and low-creativity groups: results of mixed-design ANOVA.

**Frequency band**	**Group effect**	**Group × node**
Delta	*F* = 3.781, *p* = 0.068, η^2^ = 0.174	*F* = 0.452, *p* = 0.786, η^2^ = 0.025
Theta	*F* = 0.03, *p* = 0.958, η^2^ = 0.000	*F* = 0.325, *p* = 0.819, η^2^ = 0.018
Alpha	*F* = 2.603, *p* = 0.124, η^2^ = 0.126	*F* = 0.547, *p* = 0.677, η^2^ = 0.030
Beta	*F* = 3.299, *p* = 0.086, η^2^ = 0.155	*F* = 0.661, *p* = 0.641, η^2^ = 0.035
Gamma	*F* = 0.480, *p* = 0.497, η^2^ = 0.026	*F* = 0.844, *p* = 0.492, η^2^ = 0.045

### 3.2. Phase-Lag Index Analysis

**Figure 2:**
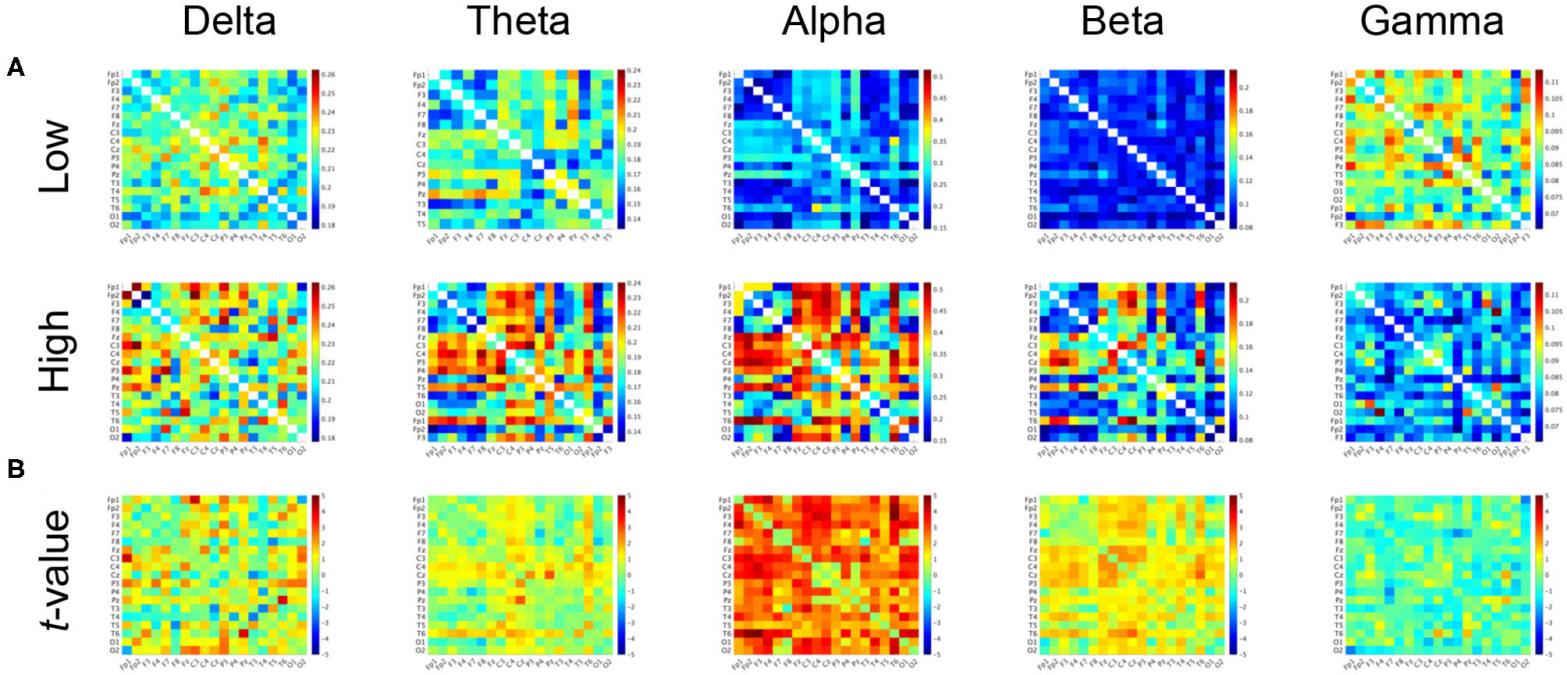
**(A)** Mean values of PLI in the low-creativity group (upper) and high-creativity group (lower). **(B)** *t*-values of the group-wise comparisons. Warm (cool) color indicates PLI values of the high-creativity group greater (less) than those of the low-creativity group. No significant differences after adjustment for FDR with *q* < 0.05 were confirmed.

**Figure 3:**
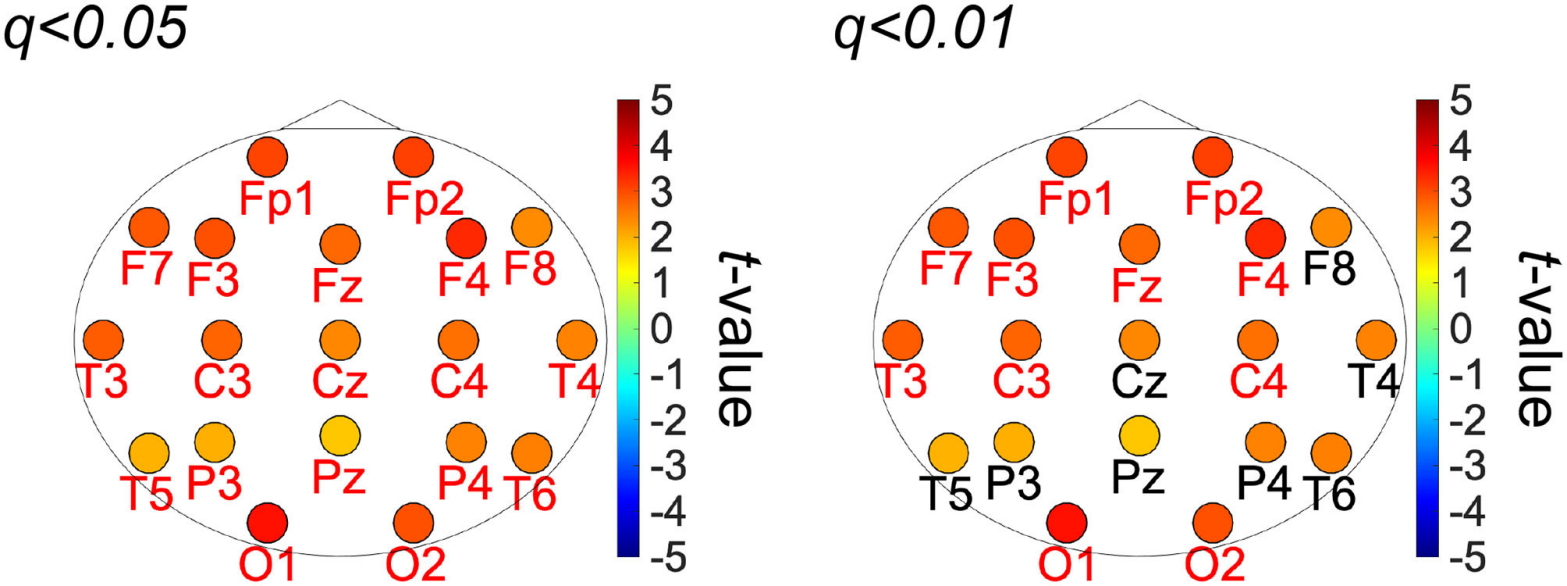
*t*-values of the topography of the PLI-based node-degree in alpha band in the group-wise comparisons. Warm (cool) color indicates the high-creativity group greater (less) than those of the low-creativity group (labels of electrodes significant after FDR: *q* < 0.01 (right), 0.05 (left) are highlighted in red).

**Figure 4:**
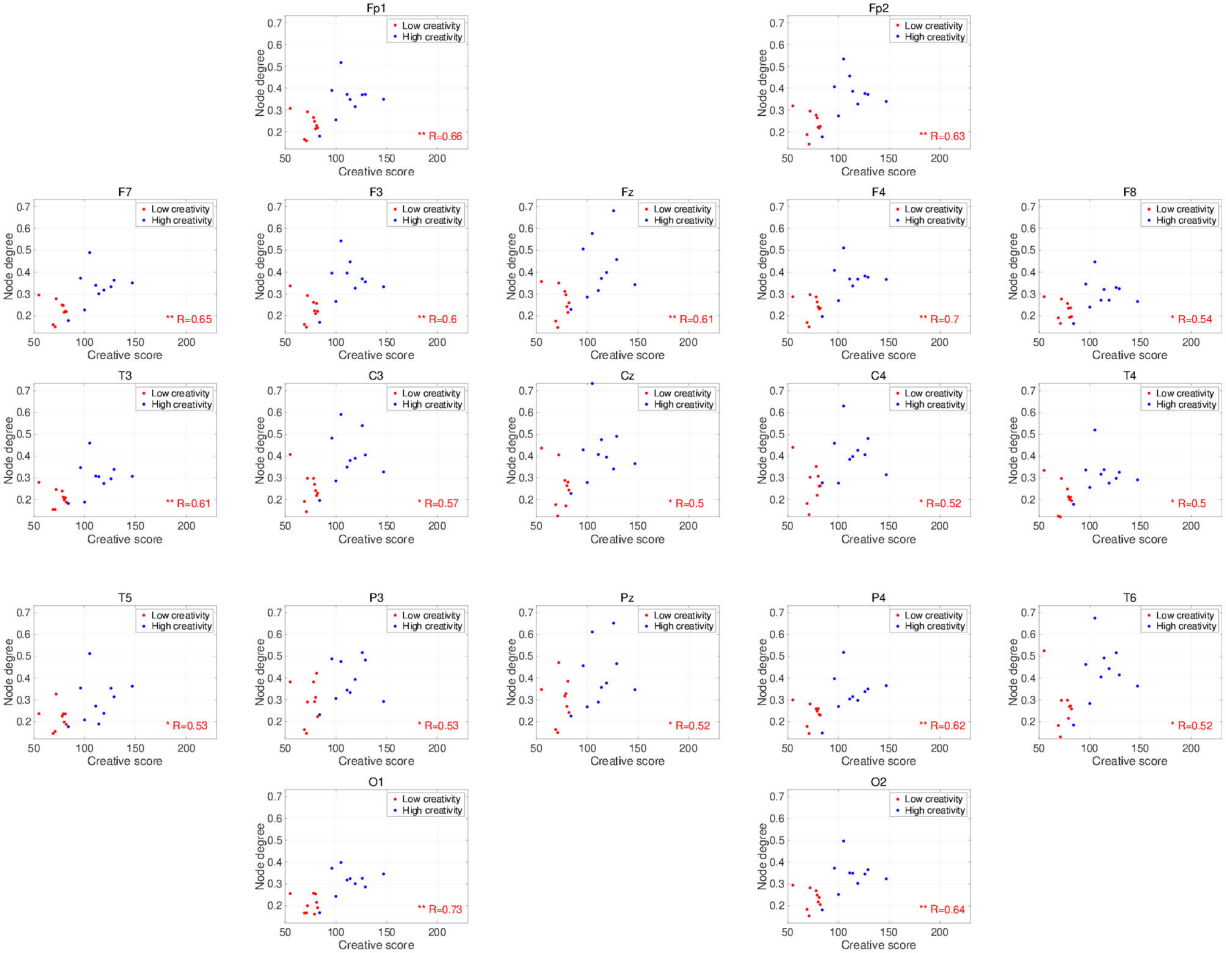
Scatter plots showing the relationships between the node degrees calculated from the PLIs in the alpha band and creativity scores. *R* is the Pearson correlation coefficient. Electrodes with significant, high correlations are labeled in red (^*^FDR criteria *q* < 0.05; ^**^q < 0.01).

**Figure 5:**
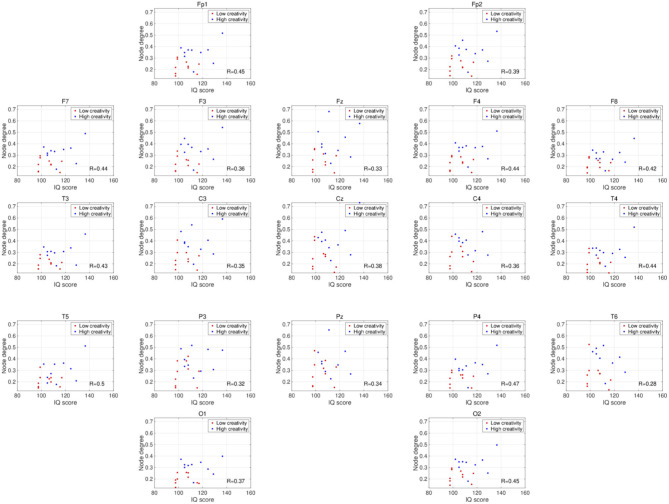
Scatter plots showing the relationships between the node degrees calculated from the PLIs in the alpha band and IQ scores. *R* is the Pearson's correlation coefficient. There are no electrodes with significant high correlations satisfying the FDR criteria *q* < 0.05.

Figure 2 shows the PLIs for high- and low-creativity groups (A) and their differences (B). The results of *t*-testing with FDR correction are presented in Figure 2B showing that pair-wise PLI values did not pass the FDR criteria (*q* < 0.05). Table 3 summarizes the results of mixed-design ANOVA for node degree, as calculated from the PLIs in each of five frequency bands. We identified a significant main effect of group in the alpha band and a significant group × node interaction effect in the beta band. The results of *post-hoc* *t*-testing with FDR correction are shown in Figure 3, which shows the node degrees that are significantly increased in the high-creativity group in the alpha band (electrode labels highlighted in red), namely all electrodes when *q* < 0.05 and those of the Fp1, Fp2, F3, F4, F7, Fz, T3, C3, C4, O1, and O2 electrodes when *q* < 0.01. To confirm the increased alpha-band PLIs observed in the high-creativity group, scatter plots of PLI-based node degrees vs. creativity scores and their Pearson correlation coefficients *R*, are shown in Figure 4. A significantly high correlation is observed at the same electrodes that showed high node degrees in the between-groups comparisons in Figure 3. In addition, the scatter plots between PLI-based node degrees in the alpha band vs. IQ scores were shown in Figure 5. Based on these results, there are no electrodes with a significantly high correlation satisfying the FDR criteria *q* < 0.05.

**Table 3:** Results of mixed-design ANOVA of PLI node degree comparing high- and low-creativity groups in each frequency band, with age and IQ as covariates.

**Frequency band**	**Group effect**	**Group × node**
Delta	*F* = 1.851, *p* = 0.190, η^2^ = 0.093	*F* = 1.182, *p* = 0.318, η^2^ = 0.062
Theta	*F* = 0.264, *p* = 0.614, η^2^ = 0.014	*F* = 1.135, *p* = 0.341, η^2^ = 0.059
Alpha	**F = 11.865, p = 0.003, η^2^ = 0.397**	*F* = 1.805, *p* = 0.134, η^2^ = 0.091
Beta	*F* = 2.129, *p* = 0.162, η^2^ = 0.106	**F = 4.056, p = 0.028, η^2^ = 0.184**
Gamma	*F* = 1.717, *p* = 0.207, η^2^ = 0.087	*F* = 1.675, *p* = 0.164, η^2^ = 0.085

*For clarity, any comparisons with p < 0.05 are shown in bold*.

## 4. Discussion and Conclusions

In this study, we examined the brain's resting-state functional connectivity, as estimated by the PLI of EEG signals in healthy older participants, and its relevance to individual creative ability, taking the level of intellectual functioning into account. The results indicate that enhanced functional connectivity in the alpha band is associated with individual creativity, irrespective of level of intellectual function. Moreover, a simple power spectrum analysis of the same EEG signals showed no relationship with individual creative ability.

Recent fMRI studies have shown that the default network plays a crucial role in creative activity (Beaty et al., 2016, 2018; Adnan et al., 2019). Beaty et al. (2014, 2018) discovered that the functional networks for divergent thinking lie in the default network and that networks that normally have an antagonistic relationship with the default network (known as executive control networks), cooperate with it during creative activity (reviewed in Beaty et al., 2016). Additionally, their analysis of resting-state fMRI revealed increased functional connectivity between the inferior prefrontal cortex and the default network in participants with high divergent-thinking ability relative to participants with low ability (Beaty et al., 2014). Beaty et al. (2018) also succeeded in generating a predictive model for creative ability by combining the whole-brain resting and task-related functional connectivities; patterns of functional connectivity in the highly creative group, consisting of frontal and parietal regions within the default, salience, and executive networks, were similar between the task-related and resting conditions. Adnan et al. (2019) reported in their fMRI study that the correlation of functional connectivity with creative activity is enhanced by aging. The results of the present study, i.e., observation of high resting-state whole brain functional connectivity in older participants having high creativity, imply the existence of a relationship between high default-network connectivity projecting among wide-spread regions and high creative activity.

The frequency-band specificity of the creativity-related functional connectivities that we observed in this study requires further discussion. Several EEG studies using power spectrum analysis have shown enhancements of neural activity in the alpha band during the performance of creative tasks (Grabner et al., 2007; Fink et al., 2009; Privodnova and Volf, 2016; Privodnova et al., 2017). Using event-related synchronization methods, Grabner et al. (2007) and Fink et al. (2009) revealed that alpha-band activity becomes significantly stronger in the right cerebral cortex during divergent thinking tasks. Other studies report creative-task–related functional connections and functional connections with a dependency on creative performance in the alpha-band (Razoumnikova, 2000; Razumnikova, 2004; Razumnikova et al., 2009; Zhou et al., 2018; Rominger et al., 2019). Additionally, Guggisberg et al. (2015) showed that the phase synchronization in the alpha band reflects global interactions among brain regions. Therefore, the alpha band PLI used in this study is considered as one of the appropriate methods to identify the functional connectivity regarding the creative activity supported by various interactions among brain regions. Furthermore, recent findings on creative ability in the aging process (Roskos-ewoldsen et al., 2008; Leon et al., 2014; Palmiero et al., 2014, 2017) have prompted studies on a possible age dependence of the specific neural processes that underlie creative activity (Privodnova and Volf, 2016; Privodnova et al., 2017; Privodnova, 2018). In particular, Privodnova and Volf (2016) and Privodnova et al. (2017) demonstrated enhancement of task-related alpha-band activity in the parieto-occipital regions in the final stage of a creative task in older participants. Therefore, the present observation of significant higher alpha-band functional connectivity estimated by the phase synchronization in higher-creativity individuals is consistent with these findings and confirms the involvement in creative activity of alpha-band neural activity/functional connectivity. However, previous studies investigated task-evoked alterations in the alpha band, whereas our study examined the resting-state EEG. As the studies of resting-state EEG, Jaušovec and Jaušovec (2000) found that alpha-band functional connectivity exhibits a negative correlation with creative ability under eyes-open, resting conditions, which seems inconsistent with the positive correlation shown here (see Figure 4). However, this inconsistency might be explained by a difference in age distribution; unlike the present study, the study by Jaušovec and Jaušovec focused on young participants in their 20s. To confirm this explanation, further evaluations of age-related changes in the functional connectivity underlying creative activity under eyes-closed, resting-state conditions are needed.

This study has some limitations. EEG signals do not necessarily reflect the neural activity directly under the electrodes, and the spatial resolution of the 19 electrodes used in this study was extremely low for identifying the complex functional connectivity structures involved in creative activity. This low spatial resolution might indicate that the PLI analysis did not identify the spatial characteristics of functional connectivity regarding creativity. Therefore, the use of neuroimaging modalities with greater spatial resolution and ability to better localize the cortical sources of signals (e.g., high density of EEG and MEG) may provide valuable additional spatial information. Moreover, recent studies showed that the indices based on graph theory, including local/global efficiencies, small-worldness, segregation, and betweenness centrality, identify the topological characteristics regarding the brain functions in both healthy and pathological conditions (Stam et al., 2009; Takahashi et al., 2017; reviewed in Bullmore and Bassett, 2011; Stam and Van Straaten, 2012; Lee and Mashour, 2018). In low, dense EEG signals used in the present study, these topological characteristics cannot be evaluated except for the node degree; however, by utilizing high spatial resolution of the high density of EEG and MEG, the relationship between the characteristics of topological whole brain network and the creativity might be revealed. The power spectrum analysis in this study did not identify the neural activity related to creative ability. However, a recent study reported a novel band ratio measure based on the power spectrum analysis (Donoghue et al., 2020). This analysis method might be utilized to evaluate the resting-state EEG signals with the complex temporal neural dynamics (Ueno et al., 2015). Additionally, to identify the mechanism for preservation of creative activity by the aging process, the analysis of creative-task-related neural activity should be extended to diverse age groups. Moreover, regarding the resting-state functional connectivity, Allaman et al. (2020) showed that the high resting-state functional connectivity enhances the performance of visual perception and motor sequence tasks. The creative activity where the default mode network plays a crucial role might exhibit the same tendency. However, to evaluate this, high spatial resolution by high dense EEG and MEG is needed to detect the region-specific brain activities and their global interaction. Therefore, our future studies will be directed toward addressing the above mentioned points.

In conclusion, the highly creative group of older individuals showed a relatively high resting-state functional connectivity in the alpha band in most electrode pairs. Despite the stated limitations, this research provides new insight into the association of functional connectivity with creativity.

## Data Availability Statement

The datasets generated for this article are not readily available because the informed consent did not include the declaration regarding publicity of clinical data. Requests to access the datasets should be directed to TT, takahash@u-fukui.ac.jp.

## Ethics Statement

The studies involving human participants were reviewed and approved by the ethics committee of the University of Fukui. The patients/participants provided their written informed consent to participate in this study.

## Author Contributions

SN, TY, TT, and HN conceived the study. SN, TY, and TT analyzed the results, drafted the main manuscript, and prepared all the figures. KU and KM conducted the experiments. All authors have reviewed and approved the manuscript.

## Conflict of Interest

The authors declare that the research was conducted in the absence of any commercial or financial relationships that could be construed as a potential conflict of interest.
