# Supplementary material for: High Phase Synchronization in Alpha Band Activity in Older Subjects With High Creativity
Source: Front Hum Neurosci. 2020 Oct 22;14:583049. doi: 10.3389/fnhum.2020.583049 (PMC7642763; doi:10.3389/fnhum.2020.583049)
Supplement: Supplementary file 1 [file Data_Sheet_1.PDF]

Supplementary Material

1 SCORES OF EACH ITEM OF S-A CREATIVITY TEST

To evaluate the creativity of each participant, we used the S-A creativity test, version C, by J.P. Guilford (Society For Creative Minds (1969)). This is an established test for the evaluation of creativity that assesses the capacity for divergent thinking. In the present study, a Japanese version of the S-A creativity test was used. The S-A test comprises three types of tasks: the first measures the ability to generate unique ways of using common objects, the second quantifies the ability to imagine desirable functions of ordinary objects, and the third measures the ability to imagine the consequences of an unforeseen event. In each task, the participants produce as many answers as possible in less than 5 min. The results of these tasks were evaluated using four criteria: fluency, flexibility, originality, and elaborate thinking. In the present study, we used the sum of these four scores to represent creative ability. To obtain two groups consisting of 10 matched subjects, participants were divided by the median of the S-A score distribution into two groups: high-creativity (high-scoring) and low-creativity (low-scoring). The total score and the scores of the four items of the S-A creativity test are represented in Table S1.

Table S1. Scores of four items of the S-A creativity test (fluency, flexibility, originality, and elaborate thinking) in high- and low-creativity groups (values are mean(SD)).

	High-creativity group ($n = 10$)	Low-creativity group ($n = 10$)
Fluency	33.4(6.1)	19.8(3.8)
Flexibility	38.8(4.1)	28.6(3.6)
Originality	14.6(4.4)	9.6(5.2)
Elaborate thinking	26.3(6.8)	13.5(3.9)
Total S-A creativity test	113.1(17.3)	71.5(11.0)

2 EVALUATION OF THE COVARIATE EFFECT OF THE INTELLIGENCE QUOTIENT AND AGE ON POWER SPECTRUM AND PHASE-LAG INDEX

2.1 Analysis of covariate effect on power spectrum

Table S2 shows the assessment of interaction effects between group and IQ/age in relative power. There is no significant interaction effect between the groups (high-creativity and low-creativity groups) and the IQ/age in relative power. In Table S3, the result of the assessment of regression between IQ/age and the relative power shows that no significant regression exist. Hence, the covariate effects of the IQ/age were not necessarily considered.

Table S2. Assessment of interaction effects between group and IQ/age in relative power. For clarity, any comparisons with $p < 0.05$ are shown in bold.

Frequency band	Group × IQ	Group × Age
delta	$F = 1.15, p = 0.343, \eta^2 = 0.133$	$F = 1.34, p = 0.291, \eta^2 = 0.152$
theta	$F = 1.619, p = 0.231, \eta^2 = 0.178$	$F = 1.55, p = 0.244, \eta^2 = 0.171$
alpha	$F = 0.784, p = 0.474, \eta^2 = 0.095$	$F = 0.752, p = 0.488, \eta^2 = 0.091$
beta	$F = 0.121, p = 0.887, \eta^2 = 0.016$	$F = 0.012, p = 0.988, \eta^2 = 0.002$
gamma	$F = 0.838, p = 0.452, \eta^2 = 0.1000$	$F = 1.774, p = 0.203, \eta^2 = 0.191$

Table S3. Assessment of the regression between IQ/age and the relative power. For clarity, any comparisons with $p < 0.05$ are shown in bold.

Frequency band	IQ	Age
delta	$F = 0.0006, p = 0.937, \eta^2 < 0.001$	$F = 0.832, p = 0.375, \eta^2 = 0.49$
theta	$F = 0.259, p = 0.618, \eta^2 = 0.016$	$F = 0.17, p = 0.685, \eta^2 = 0.011$
alpha	$F = 0.028, p = 0.868, \eta^2 = 0.002$	$F = 0.258, p = 0.618, \eta^2 = 0.016$
beta	$F = 0.207, p = 0.656, \eta^2 = 0.013$	$F = 0.006, p = 0.940, \eta^2 < 0.001$
gamma	$F = 1.74, p = 0.206, \eta^2 = 0.098$	$F = 3.78, p = 0.07, \eta^2 = 0.191$

2.2 Analysis of covariate effect on phase-lag index

Table S4 shows the assessment of the interaction effects between the groups and the IQ/age in the node degree of phase-lag index (PLI). There are significant interaction effects of group × IQ at the theta and beta bands and the significant interaction effects of group × age at the beta band. In Table S5, the result of assessment of regression between IQ/age and the node degree showed significant regression with IQ at the theta and beta bands. However, these regression slopes were not parallel between high-creative and low-creative groups shown in $p < 0.05$ the Table S4. Hence, the covariate effects of IQ/age were not necessarily considered.

Table S4. Assessment of interaction effects between group and IQ/age in PLI node degree. For clarity, any comparisons with $p < 0.05$ are shown in bold.

Frequency band	Group × IQ	Group × Age
delta	$F = 1.581, p = 0.238, \eta^2 = 0.174$	$F = 0.410, p = 0.671, \eta^2 = 0.052$
theta	$\mathbf{F = 5.765, p = 0.014, \eta^2 = 0.435}$	$F = 0.011, p = 0.989, \eta^2 = 0.002$
alpha	$F = 2.323, p = 0.132, \eta^2 = 0.237$	$F = 1.789, p = 0.201, \eta^2 = 0.193$
beta	$\mathbf{F = 19.949, p < 0.001, \eta^2 = 0.727}$	$\mathbf{F = 15.018, p < 0.001, \eta^2 = 0.667}$
gamma	$F = 2.234, p = 0.141, \eta^2 = 0.23$	$F = 2.359, p = 0.129, \eta^2 = 0.239$

Table S5. Assessment of regression between IQ/age and PLI node degree. For clarity, any comparisons with $p < 0.05$ are shown in bold. The IQ/age and frequency band pairs where the significant interaction effects were confirmed in table S4, are underlines.

Frequency band	IQ	Age
delta	$F = 2.415, p = 0.140, \eta^2 = 0.131$	$F = 0.383, p = 0.544, \eta^2 = 0.023$
theta	$\underline{\mathbf{F = 12.117, p = 0.003, \eta^2 = 0.431}}$	$F = 0.002, p = 0.963, \eta^2 < 0.001$
alpha	$F = 0.585, p = 0.455, \eta^2 = 0.035$	$F = 0.509, p = 0.486, \eta^2 = 0.031$
beta	$\underline{\mathbf{F = 4.501, p = 0.05, \eta^2 = 0.22}}$	$\underline{F = 1.255, p = 0.279, \eta^2 = 0.073}$
gamma	$F = 2.32, p = 0.147, \eta^2 = 0.127$	$F = 2.25, p = 0.153, \eta^2 = 0.123$

REFERENCES

Society For Creative Minds (1969). Manual of SA creativity test
